# Helpful Criteria When Implementing NGS Panels in Childhood Lymphoblastic Leukemia

**DOI:** 10.3390/jpm10040244

**Published:** 2020-11-26

**Authors:** Nerea Vega-Garcia, Rocío Benito, Elena Esperanza-Cebollada, Marta Llop, Cristina Robledo, Clara Vicente-Garcés, Javier Alonso, Eva Barragán, Guerau Fernández, Jesús M. Hernández-Sánchez, Marta Martín-Izquierdo, Joan Maynou, Alfredo Minguela, Adrián Montaño, Margarita Ortega, Montserrat Torrebadell, José Cervera, Joaquín Sánchez, Antonio Jiménez-Velasco, Susana Riesco, Jesús M. Hernández-Rivas, Álvaro Lassaletta, José María Fernández, Susana Rives, José Luis Dapena, Manuel Ramírez, Mireia Camós, on behalf of the Group of Leukemia of the Spanish Society of Pediatric Hematology and Oncology (SEHOP)

**Affiliations:** 1 Hematology Laboratory, Hospital Sant Joan de Déu Barcelona, Passeig Sant Joan de Déu 2, 08950 Esplugues de Llobregat, Barcelona, Spain; ecebollada@sjdhospitalbarcelona.org (E.E.-C.); cvicenteg@fsjd.org (C.V.-G.); mtorrebadell@sjdhospitalbarcelona.org (M.T.); 2 Leukemia and other Pediatric Hemopathies, Developmental Tumors Biology Group, Institut de Recerca Hospital Sant Joan de Déu, Santa Rosa 39-57, 08950 Esplugues de Llobregat, Barcelona, Spain; Srives@sjdhospitalbarcelona.org (S.R.); jdapena@sjdhospitalbarcelona.org (J.L.D.); 3 IBSAL, IBMCC, CIC, Universidad de Salamanca-CSIC, 37008 Salamanca, Spain; beniroc@usal.es (R.B.); jesus807@gmail.com (J.M.H.-S.); marta.martini@usal.es (M.M.-I.); adrianmo18@gmail.com (A.M.); 4 Molecular Biology Unit, Hospital Universitario y Politécnico La Fe, 46026 Valencia, Spain; llop_margar@gva.es (M.L.); barragan_eva@gva.es (E.B.); 5 Unidad de Tumores Sólidos Infantiles, Instituto de Investigación de Enfermedades Raras, Instituto de Salud Carlos III, Majadahonda, 28222 Madrid, Spain; c.robledo@externos.isciii.es (C.R.); fjalonso@isciii.es (J.A.); 6 Centro de Investigación Biomédica en Red de Enfermedades Raras, Instituto de Salud Carlos III (CB06/07/1009; CIBERER-ISCIII), 28029 Madrid, Spain; 7 Molecular and Genetics Medicine Section, Hospital Sant Joan de Déu, Barcelona, Passeig Sant Joan de Déu 2, 08950 Esplugues de Llobregat, Barcelona, Spain; gfernandezi@sjdhospitalbarcelona.org (G.F.); jmaynou@sjdhospitalbarcelona.org (J.M.); 8 Institut de Recerca Hospital Sant Joan de Déu, Santa Rosa 39-57, 08950 Esplugues de Llobregat, Barcelona, Spain; 9 Immunology Service, Virgen de la Arrixaca Clinic University Hospital, Biomedical Research Institute of Murcia (IMIB-Arrixaca), El Palmar, 30120 Murcia, Spain; alfredo.minguela@carm.es; 10 Department of Hematology, University Hospital Vall d’Hebron, University Autònoma of Barcelona (UAB), 08035 Barcelona, Spain; maguiortega@yahoo.es; 11 Centro de Investigación Biomédica en Red de Enfermedades Raras (CIBERER), Instituto de Salud Carlos III, 28029 Madrid, Spain; 12 Hematology Service and Genetics Unit, Hospital de La Fe, 46026 Valencia, Spain; cervera_jos@gva.es; 13 Hematology Department, Hospital Reina Sofía, IMIBIC, University of Córdoba, 14004 Córdoba, Spain; joaquin.sanchez@cheerful.com; 14 Hematology and Hemotherapy Laboratory, Hospital Carlos Haya, 29010 Málaga, Spain; antoniof.jimenez.sspa@juntadeandalucia.es; 15 Pediatric Service, Hospital Universitario de Salamanca-IBSAL, 37007 Salamanca, Spain; sriesco@saludcastillayleon.es; 16 Hematology Service, Hospital Universitario de Salamanca-IBSAL-USAL, 37007 Salamanca, Spain; jmhr@usal.es; 17 Department of Pediatric Hematology & Oncology, Hospital Universitario Niño Jesús, 28009 Madrid, Spain; lassaalvaro@yahoo.com; 18 Pediatric Oncohematology Unit, Hospital de La Fe, 46026 Valencia, Spain; chemafer@me.com; 19 Paediatric Haematology and Oncology Department, Hospital Sant Joan de Déu Barcelona, University of Barcelona, 08950 Barcelona, Spain; 20 Hematology Laboratory, Hospital Niño Jesús, 28009 Madrid, Spain; manuel.ramirez@salud.madrid.org

**Keywords:** next-generation sequencing, NGS-targeted panel, childhood acute lymphoblastic leukemia

## Abstract

The development of Next-Generation Sequencing (NGS) has provided useful diagnostic, prognostic, and therapeutic strategies for individualized management of B-cell precursor acute lymphoblastic leukemia (BCP-ALL) patients. Consequently, NGS is rapidly being established in clinical practice. However, the technology’s complexity, bioinformatics analysis, and the different available options difficult a broad consensus between different laboratories in its daily routine introduction. This collaborative study among Spanish centers was aimed to assess the feasibility, pros, and cons of our customized panel and other commercial alternatives of NGS-targeted approaches. The custom panel was tested in three different sequencing centers. We used the same samples to assess other commercial panels (Oncomine^TM^ Childhood Cancer Research Assay; Archer^®^FusionPlex^®^ ALL, and Human Comprehensive Cancer Panel GeneRead Panel v2^®^). Overall, the panels showed a good performance in different centers and platforms, but each NGS approach presented some issues, as well as pros and cons. Moreover, a previous consensus on the analysis and reporting following international guidelines would be preferable to improve the concordance in results among centers. Our study shows the challenges posed by NGS methodology and the need to consider several aspects of the chosen NGS-targeted approach and reach a consensus before implementing it in daily practice.

## 1. Introduction

Acute lymphoblastic leukemia (ALL) is the most common pediatric cancer and the primary cause of death related to cancer in childhood [1,2,3]. Pediatric ALL’s survival rates have improved significantly due to better prognostic stratification and risk-directed therapies [4]. However, a relevant proportion of patients relapse, and short and long-term side effects are the main concern in survivors [5]. Thus, more personalized medicine is still needed. The recent advent of Next-Generation Sequencing (NGS) technologies has provided the methodology to find useful biomarkers to tailor treatment and manage patients individually. NGS provides the potential for increased throughput, sensitivity, and quantification of variant allelic frequencies (VAF) compared to Sanger sequencing [6]. Importantly, each NGS step can influence the results: DNA extraction, library preparation, sequencing technologies, quality controls, bioinformatic filtering, biological interpretation in the clinical context, and reporting. Moreover, the existence of several NGS platforms allows the comparison of different approaches but also makes standardization indispensable to provide homogeneous and reliable results. Global analyses of the entire genome (Whole-Genome Sequencing, WGS) or exome (Whole-Exome Sequencing, WES) are available in research. Still, few of these approaches are offered by clinical laboratories to guide routine clinical practice: they remain expensive, time-consuming, and laborious [7]. Therefore, we need to incorporate easier and cost-effective targeted sequencing approaches into clinical practice [8]. Targeted approaches require design, library preparation protocols, and enrichment assays to achieve accurate variant calling that enables us to define relevant causative variants, but they offer better base-pair coverage, running times, costs, and easier dataset handling than WGS and WES [9]. Optimal panels should provide useful information on diagnosis, prognosis, and potential therapeutic targets and remain easy to use in daily routine settings. Nowadays, there are a plethora of different custom-NGS panels developed by research groups and commercially available panels. Thus, although several options for genomic patients profiling are currently available, it is important to perform a detailed study of their scope before choosing them. Finally, the appropriate analysis, interpretation of the pathogenicity, and integration with other molecular findings remain a major challenge, especially for novel variants, those of uncertain significance, as well as for incidental findings [10].

This study is a collaborative project of the Biological Committee of the Group of Leukemia of the Spanish Society of Pediatric Hematology and Oncology (SEHOP). We aimed to test four NGS panels (one custom panel and three commercial panels) for their application in clinical routine, in the setting of the diagnosis of B-cell precursor ALL (BCP-ALL). We dissect each panel and discuss their design, analysis, and applicability related to BCP-ALL. Our study also examines the challenges that lay ahead before introducing and harmonizing the NGS-targeted approaches in clinical routine for childhood BCP-ALL diagnosis. The present study may offer helpful criteria for hematological genetic laboratories when implementing new NGS panels.

## 2. Materials and Methods

### 2.1. Ethical Issues

According to the national and international guidelines, the study strictly followed the ethical standards and the Declaration of Helsinki. All the samples were stored in the Biobank of Hospital Sant Joan de Déu (HSJD) and, according to our Ethics Committee. Samples were used after written informed consent was obtained from the patients or their legal guardians.

### 2.2. Design of the Study

The current study aimed to test on a pilot cohort of pediatric BCP-ALL patients, the pros and cons of each panel, and highlight critical points in NGS panel choice to improve BCP-ALL diagnostics. The following factors were considered: (1) the same assay was tested in different centers; (2) the same samples were tested on various assays; (3) to reproduce real-life faithfully, no previous consensus in bioinformatics pipelines and reporting was achieved before the study.

Thus, four NGS-targeted panels were tested: (1) the BCP-ALL custom panel; (2) Archer^®^ FusionPlex^®^ ALL kit (ArcherDX, Boulder, CO, USA); (3) Oncomine^TM^ Childhood Cancer Research Assay (OCCRA) (ThermoFisher Scientific, Waltham, MA, USA); and (4) Human Comprehensive Cancer GeneRead Panel v2^®^ (Qiagen, Hilden, Germany), using Illumina (San Diego, CA, USA) and Ion Torrent (Gilford, NH, USA) platforms.

To assess the performance of the different panels, DNA and RNA from 4 BCP-ALL pediatric patients (see Supplementary Material for detailed information) were distributed. Those samples have been previously diagnosed and genetically characterized in center 1 (HSJD) by conventional methods. To evaluate the harmonization between different centers in NGS testing implementation, the samples were sent to center 2: Hospital Universitario de Salamanca (HUS); center 3: Hospital Niño Jesús (HNJ)-Instituto de Salud Carlos III (ISCIII); and center 4: Hospital La Fe (HLF). The main characteristics of patients are detailed in Supplementary Table S1.

The workflow of samples, panels, and platforms is shown in Supplementary Figure S1.

### 2.3. NGS-Targeted Panels Included in the Study

In this study, we have examined the performance of four NGS-targeted panels. To reproduce real-life faithfully in clinical laboratories, the panel selection was made following different criteria in individual centers, including NGS panel target design, equipment, and human and economic resources. We started our study by dissecting the currently available information about panel specifications, such as technical specifications and genes included (see below).

The genes present in the four panels are listed in Supplementary Tables S2–S5.

#### 2.3.1. BCP-ALL Custom Panel

The BCP-ALL custom panel was designed to screen for single nucleotide variants (SNVs) and copy number variant (CNV). We selected 69 genes of diagnostic, prognostic, or therapeutic interest recurrently identified in BCP-ALL according to the literature. Details on the probe design are provided in Supplementary Methods.

#### 2.3.2. Archer^®^ FusionPlex^®^ ALL Kit

The Archer^®^ FusionPlex^®^ ALL Kit uses 506 gene-specific primers (GSPs) that target 81 genes commonly mutated in ALL. This panel uses the Anchored Multiplex PCR (AMP), which allows identifying fusions with both known and unknown partners. For the library generation, two different protocols were applied following the manufacturer’s specification depending on the sequencing platform used (Illumina or Ion Torrent). Details on the performance are provided in Supplementary Methods.

#### 2.3.3. Oncomine^TM^ Childhood Cancer Research Assay (OCCRA)

The Oncomine^TM^ Childhood Cancer Research Assay (Ion Torrent, ThermoFisher) is a pan-pediatric cancer panel. It includes the analysis in hot spots regions of 82 genes, the full coding sequence of 44 targets, and the CNV variation in 24 genes. It can also detect rearrangements of 88 drivers and quantify the expression of 9 targets when RNA is analyzed. Details on the performance are provided in Supplementary Methods.

#### 2.3.4. Human Comprehensive Cancer GeneRead Panel v2^®^

This commercial panel includes 160 genes frequently mutated in cancer. Details on the performance are provided in Supplementary Methods.

### 2.4. Sequencing and Variant Data Analysis

Sequencing was performed using Illumina (San Diego, CA, USA) and Ion Torrent (Gilford, NH, USA) platforms depending on the panel manufacturer’s specifications. Further details on sequencing and bioinformatics pipelines are provided in Supplementary Methods.

The main criteria for manual variant prioritization in center 1 were: (1) exclusion of variants with a population frequency (Minor Allele Frequency, MAF) of ≥1% in the 1000 Genomes database; (2) exclusion of synonymous variants; (3) inclusion of only exonic variants (intronic regions were set aside for a second reanalysis); and (4) calls with a VAF ≥ 5% and minimum coverage of 50× per variant, which were considered to be of enough quality to be interpreted and potentially reported. The Integrative Genomics Viewer (IGV) (Broad Institute and the Regents of the University of California) version 2.5.011 was used to visually assess the authenticity of individual calls and exclude potential artifacts. To prioritize variants in centers 2, 3, and 4, synonymous variants, noncoding variants, or variants present in population databases (including dbSNP144, the 1000 genomes Project, ExAC, ESP 6500, and in-house database) at a MAF ≥ 1% were discarded. Variants recurrently observed and suspected of being sequencing errors by visual inspection on the IGV version 2.3.68 were removed. However, all the known hotspot mutations and the variants described in the Catalogue of Somatic Mutations in Cancer database (COSMIC82 database) and/or in ClinVar were rescued. To elucidate the effects of variants with no clear clinical significance, the PolyPhen-2, SIFT, and Mutation Taster web-based platforms were used. The mutations with deleterious and/or probably damaging effects were also considered as somatic mutations, whereas the remaining variants were considered candidate somatic mutations.

For CNV detection, the mean coverage depth of an individual exon target of a sample was first normalized to the total amount of the DNA template loaded onto sequencing flow cells, based on this sample’s total reads. The mean coverage of each target from the reference samples obtained was used as the reference for a specific exon. A reference coverage profile for a normal sample was generated. Finally, the normalized coverage of each exon of a test sample was compared to the mean coverage of the same target in the reference file generated above [9].

## 3. Results

The four samples were sequenced using the custom panel and three commercially available NGS panels. Results from DNA sequencing are shown in Supplementary Table S6.

The previously characterized mutations with the custom panel were found with the commercial panels if the design covered their genomic location.

### 3.1. Dissection of the NGS Panels

#### 3.1.1. BCP-ALL Custom Panel

**Figure 1:**
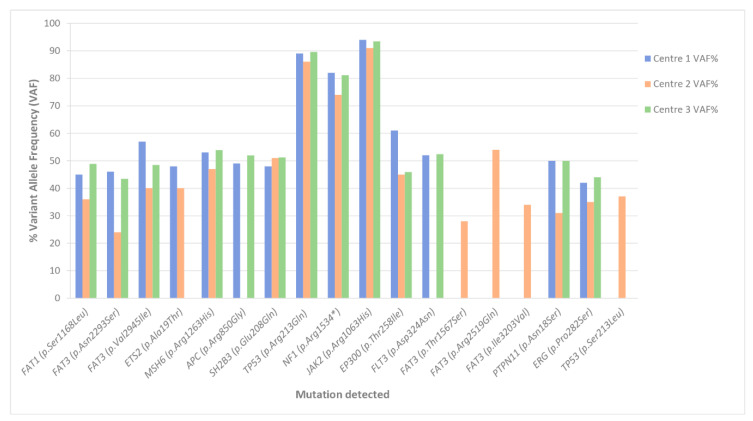
Variant allele frequency of variants detected by each center in the four common samples analyzed, using the B-cell precursor acute lymphoblastic leukemia (BCP-ALL) custom panel.

Our BCP-ALL custom panel was intended to search for SNVs and CNVs by using DNA as a template. All samples passed the quality controls. The final molarity loaded on the NextSeq flow cell was 1.6 pM. NextSeq cluster densities achieved for the BCP-ALL custom panel libraries were as expected, with a median of 5.67 million reads and 93.6% on-target coverage. To assess the reproducibility, the four samples were sequenced independently in three different centers. Sequencing metrics, variant detection, and VAF were highly reproducible between centers, and the results were in concordance with those obtained by other methodologies (Figure 1 and Supplementary Table S6). We compared all variants obtained before data curation, so we included in the comparison all the different variants detected, including Single Nucleotide Polymorphism (SNPs).

Overall, the BCP-ALL custom panel detected a mean of 0.75 variations per patient in 13 genes. All were SNVs (*n* = 3), and neither small frameshift insertions nor deletions were found.

Those variants with VAF around 50% were studied in matched samples obtained in complete remission (CR) with negative minimal residual disease (MRD) (and thus considered non-somatic): *FAT1*, *FAT3*, and *ETS2* in sample 1; *MSH6*, *APC,* and *SH2B3* in sample 2; *EP300*, *JAK2*, *FLT3*, and *FAT3* in sample 3, and *PTPN11* and *ERG* in sample 4. Overall, only *TP53* and *NF1* in sample 2 were confirmed as somatic mutations and were detected by all centers. Furthermore, *NF1* variant was detected in homozygosis; this fact is probably related to the loss of genetic material evidenced in the karyotype (see Supplementary Table S1).

**Figure 2:**
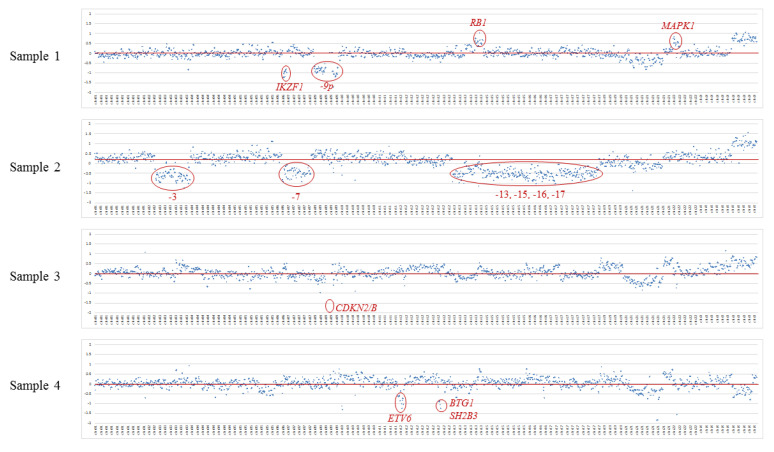
Copy number variation (CNV) analysis by massively parallel sequencing. Log2 ratios of normalized mean coverage of each target were plotted against the reference. The *x*-axis is the corresponding targets in the panel, plotted by relative genome order, and the *y*-axis shows the log2 ratio of mean coverage of testing to that of reference. CNVs were called using fixed thresholds representing the minimum log2 ratio for gains (0.55) and maximum log2 ratio for losses (−0.55).

CNV analysis confirmed the presence of alterations previously identified by karyotype and/or Multiplex Ligation-Dependent Probe Amplification (MLPA). Sample 2 showed the loss of chromosomes 3, 7, 13, 15, 16, and 17, consistent with the corresponding monosomies and the low hypodiploidy observed in the karyotype and the pattern obtained by the MLPA, P181, and P182 kits. Additionally, the custom panel detected a focal deletion of *IKZF1*, loss of 9p, and gains of *RB1* and *MAPK1* in sample 1, loss of *CDKN2A*/*B* in sample 3, and loss of *ETV6*, *BTG1,* and *SH2B3* genes in sample 4 (Figure 2). Most of those abnormalities had been previously identified by using the MLPA P335, P181, and P182 kits.

#### 3.1.2. Archer^®^ FusionPlex^®^ ALL Kit

This is a panel intended to detect gene fusions using RNA as a template. In this study, the panel was sequenced on two different platforms, Illumina and Ion Torrent.

We confirmed that the total number of RNA reads per sample was not significantly different when the Archer^®^ FusionPlex^®^ ALL kit was run on two different platforms (Illumina or Ion Torrent) (*p* = 0.8).

The detection of the rearrangements was concordant between platforms in 4 out of 7 rearrangements detected. Sample 1 was known to harbor the t(9;22)(q34;q11), which was detected by the panel regardless of the platform. Sample 2 presented an *LMO1*-*RIC3* fusion identified by both platforms. A *BCL11B* rearrangement was also detected when sequenced with an Illumina platform but was not found on Ion Torrent. On the other hand, an *IKZF1* exon skipping was called by Ion Torrent but was not found when the panel was sequenced with Illumina equipment. No rearrangements were found in sample 3 harboring a dic(9;20)(p11-13;q11), although a *PAX5* fusion was suspected. *ETV6*-*RUNX1* rearrangement, previously confirmed by routine diagnostic techniques, was detected in sample 4, and a *GGYF2*-*PBX1* fusion was also identified in this sample by both platforms. When this panel was sequenced on an Ion Torrent, a *RUNX1*-*ETV6* fusion was also identified in sample 4.

Although the panel’s main purpose is to detect fusion genes, it is possible to detect SNVs when using an Illumina platform and analyzed using the Archer^®^ analysis platform. In this case, we found a variant in *SH2B3* in sample 2 and another variant in *PTPN11* in sample 4. When the samples are sequenced on Ion Torrent and analyzed using IonReporter, the analysis of SNVs is not offered.

#### 3.1.3. Oncomine^TM^ Childhood Cancer Research Assay (OCCRA)

Among the tested panels, this was the only panel able to detect SNVs and fusion genes by analyzing both DNA and RNA. Overall, OCCRA panel detected a mean of 1.75 SNVs per patient in 6 genes and 1 rearrangement in two patients.

This panel did not identify any SNV in sample 1; however, it detected a *BCR*-*ABL1* fusion. Several variants were found in sample 2 in *APC*, *SH2B3*, *TP53,* and *NF1,* but no rearrangement was found. For sample 3, the panel identified a SNV in *JAK2* and *FLT3* and, as in sample 2, no rearrangement was found. Finally, a *TP53* variant and *ETV6*-*RUNX1* fusion were found in sample 4.

#### 3.1.4. Human Comprehensive Cancer GeneRead Panel v2^®^

The Human Comprehensive Cancer GeneRead Panel v2^®^ analyzes DNA to search for SNVs. This panel showed mutations in all the samples analyzed, with a mean of 2.75 variants per patient in 10 genes.

Specifically, this panel showed mutations in *PIK3R1*, *PALB2,* and *ASXL1* genes in sample 1. Sample 2 harbored variants in *MSH*, *APC,* and *TP53*. In sample 3, the panel identified SNVs in *JAK2*, *EP300,* and *TP53*. Sample 4 presented mutations in *PTPN11* and *TP53*.

### 3.2. Panel Dissection

**Figure 3:**
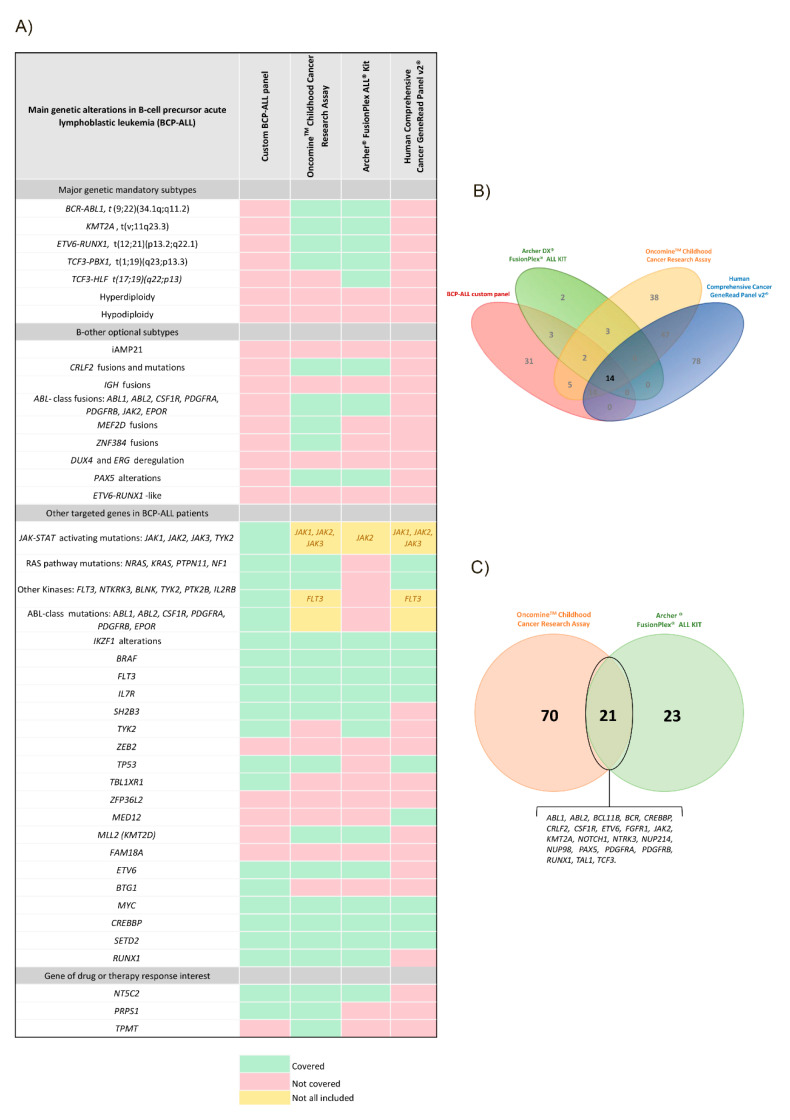
Gene content of the different targeted panels. (**A**) Approach of the different panels to assess the main genetic alterations in BCP-ALL. (**B**) Overlapping gene content in the different Next Generation Sequencing-targeted compared panels. (**C**) Overlapping fusion content in the Oncomine^TM^ Childhood Research Assay and Archer^®^ FusionPlex^®^ ALL kit.

Main genetic alterations with clinical relevance for pediatric BCP-ALL diagnosis, overlapping gene content, and overlapping fusion content among panels are represented in Figure 3A–C, respectively.

All four panels had fourteen overlapping genes for SNV (*BRAF*, *CREBBP*, *CRLF2*, *EZH2*, *FLT3*, *IL7R*, *JAK1*, *JAK2*, *JAK3*, *KRAS*, *NF1*, *NRAS*, *PAX5,* and *PTPN11*). These 14 genes are known to be involved in ALL. The remaining genes present in the different panels were useful for different aims. Three of the four panels were specifically designed to improve childhood BCP-ALL diagnostics, as the genes included were relevant in pediatric cancer or ALL. The custom BCP-ALL panel was the most specific to search for mutations in pediatric ALL (Figure 3A) as it was designed for this project with this objective but did not cover gene fusions. Archer^®^ FusionPlex^®^ ALL kit was focused on fusion gene identification in ALL. OCCRA was designed to identify mutations and fusions in pediatric cancer, including solid tumors and hematological malignancies. Finally, the Human Comprehensive Cancer GeneRead Panel v2^®^ had a broader approach, as it was conceived for the study of different types of cancer, mostly focused on adult cancer, but only detected mutations. Thus, two of the four panels, OCCRA and Archer^®^ FusionPlex^®^ ALL kit, were able to detect fusion genes. A total of 21 fusions were common among the panels. However, the design of all four panels did not include the study of highly relevant fusion genes in the BCP-ALL diagnostic: the custom BCP-ALL panel and Human Comprehensive Cancer GeneRead Panel v2^®^ did not cover gene fusions, while Archer Fusion Plex comprised some of them (Figure 3A).

#### 3.2.1. SNV Detection

Filtered Variant Call Format (VCF) obtained from the different in-house analysis for custom BCP-ALL panel or software analysis for commercial panels were compared. The variants called in each panel are shown in Supplementary Table S7. After variant calling, a similar number of variants per patient were called by the different panels. However, some differences were observed in the gene variant detection since the genes covered in each panel are different.

When focusing on the more common genes for SNV detection between the panels, variant calling and filtering was comparable. Correlation of the VAFs detected by each panel showed a high concordance level between panels for SNV detection.

Only the variant reporting process showed small discordances among centers for the custom BCP-ALL panel (described below). Thus, although similar variants and VAF were reported individually by different sequencing centers, some differences in reported variants were found (Figure 1 and Supplementary Table S6). In sample 1, the *ETS2* variant found by centers 1 and 2 was not reported by center 3, as it was considered a polymorphism after finding a MAF > 1% in 8 different databases. In sample 2, center 2 missed the *APC* variant due to the low depth of coverage of this region (only 23 total base reads). In sample 3, three *FAT3* variants were reported by center 2, but not by centers 1 and 3, as these variants were considered polymorphisms and filtered out during variant prioritization. Similarly, centers 1 and 3 reported a variant in *FLT3* that was not reported by center 2, as it was considered a polymorphism, being reported in 2 databases with a MAF > 1% and presenting only one entry in the COSMIC database. The *TP53* variant in sample 4 was only reported by center 2. In this case, discordance was attributed to differences in the transcript choice. The centers used two different transcripts, which had a major effect on the ultimate variant annotation. Thus, center 2 used transcript NM_001126117.1 to annotate this variant, obtaining an exonic missense variant in *TP53* (p.(Ser213Leu); c.638C > T). In contrast, center 1 and 3 used transcript NM_000546.5, thus obtaining an intronic variant (c.993 + 309C > T), which was filtered out during the variant prioritization.

There were no differences between commercial panel reporting because the same software analysis was used.

#### 3.2.2. CNV Detection

Since only the custom panel was designed to detect CNVs, no comparison between panels was performed.

#### 3.2.3. RNA Fusion Detection

Our study showed that both assays that covered fusion detection, the Archer^®^ FusionPlex^®^ ALL kit and OCCRA, successfully detected fusions with known partners, previously identified by RT-PCR.

The total number of RNA reads did not differ significantly between the Archer^®^ FusionPlex^®^ ALL kit and the OCCRA panel (*p* = 0.3). The detection of rearrangements was concordant in 2 out of 4 samples. No rearrangements were found in sample 3, harboring a dic(9;20)(p11-13;q11). The *ETV6*-*RUNX1* rearrangement, previously confirmed by routine diagnostic techniques, was detected in sample 4 by both commercial panels. On the other hand, sample 1 was known to harbor the t(9;22)(q34;q11)/*BCR-ABL1*, which was detected by both commercial panels, although the number of *BCR-ABL1* transcript reads differed between platforms (a mean of 5067 reads on Archer^®^ FusionPlex^®^ ALL kit vs. a mean of 245,820 reads on OCCRA). The Archer^®^ Fusionplex^®^ ALL kit found the *LMO1-RIC3* fusion in sample 2 in two independent runs (centers 2 and 4), whereas the OCCRA panel did not detect this transcript, as it is not included in its design.

All RNA results are shown in Supplementary Table S8.

## 4. Discussion

The use of NGS technologies has dramatically increased in clinical laboratories, as therapeutic protocols demand the screening of multiple genetic abnormalities with diagnostic, stratification, or therapeutic relevance. Besides, NGS has helped in the identification of targets for MRD follow-up. However, choosing the optimal NGS strategy for daily routine can be difficult. Genome-wide NGS approaches provide a high amount of information and are progressively cheaper, but the complexity in their analysis hampers their use in daily routine. NGS-targeted panels, in contrast, may represent a good option to implement in clinical practice. In our collaborative study, we assessed different currently available NGS-targeted approaches to apply in the setting of pediatric BCP-ALL diagnosis. We designed a custom panel and assessed its performance and that of three different NGS commercial panels. Overall, we demonstrated similar results for the different platforms and panels and confirmed their utility in a clinical context. However, some issues should be considered before implementing NGS in clinical practice in childhood ALL.

### 4.1. Panel Discussion

NGS panel target design depends on the panel’s intended use, as they can be designed with different scope. Collectively, the purpose and applicability of the different targeted panels tested were compared for their use in clinical laboratories (Supplementary Figure S2). The four panels had in common the presence of alterations described in BCP-ALL. The custom BCP-ALL panel had advantages in detecting SNV and CNV in selected genes related to pediatric BCP-ALL. OCCRA combined SNV and fusion detection and was focused on pediatric cancer-related genes. Archer^®^ FusionPlex^®^ ALL, based on its semi-anchored PCR, allowed to identify known fusion genes and discover new fusion partners, which may also be useful for research applications. On the other hand, the Human Comprehensive Cancer GeneRead v2^®^ panel screened a large number of genes.

In our study, the four sequencing centers showed successful performance and concordance in results, not only in terms of variant detection but also in the VAFs obtained for each sample. However, different platforms showed some differences in fusion detection using the same commercial panel, probably due to the differences in the analytical software provided by the different companies. These results highlight the necessity of verifying the results by other methods. Our data confirm our custom panel’s reproducibility in different centers with the same platform, but not the full comparability of NGS platforms with different chemistries using the same panel. As used in this study, different NGS panels and platforms demonstrated comparable performance in the detection of somatic variants from DNA samples across multiple genes and a wide range of VAFs.

In terms of hands-on, all the NGS-targeted panels required time due to their extensive protocols consisting of various PCR steps. Our study tested one hybridization capture targeted panel (BCP-ALL custom panel) and 3 amplicon-based targeted panels (OCCRA, Archer^®^ FusionPlex^®^ ALL, and the Human Comprehensive Cancer GeneRead v2^®^). In general, amplicon-based panels are less time consuming than capture-based panels, shortening the time to get the results. Therefore, it is important to consider this point before choosing a panel to implement in daily routine. In terms of cost-effectiveness, generally, NGS panels are the most widely used tool for clinical applications mainly for cost-effectiveness reasons, as they allow a comprehensive study of the disease. However, different tests are still needed to identify all relevant genetic abnormalities contemplated in the therapeutic protocols.

Finally, it was not the scope of our study to demonstrate the clinical impact of the panels analyzed. Instead, we focused on each approach’s feasibility and performance and used a very limited number of cases. We selected four patients harboring already known clinically relevant abnormalities in terms of risk stratification and the prognostic impact such as *TP53* and *NF1* variants and *BCR-ABL1* and *ETV6-RUNX1* rearrangements. The commercial panels targeting RNA detected the rearrangements successfully, and all the DNA-based NGS panels correctly identified the variants, provided that adequate coverage of those regions was achieved (Supplementary Table S7).

Some factors may limit our study. A higher number of samples would be desirable. We also did not compare the different bioinformatics processes used by the different assays because of the significant differences in the panel design, library preparation, and sequencing platform. Thus, a truly fair comparison was not possible in our study, although it could be technically meaningful. Our study did not intend to carry out a technical validation of the panels; rather, we took the approach of a qualitative comparison to dissect the panels as a first step before choosing a panel. We also planned this study as the basis to harmonize the implementation of an NGS panel for the study of pediatric BCP-ALL in different centers in our country. However, a thorough validation of panels, including commercial assays, is needed before their implementation in clinical diagnostics.

### 4.2. Implementing NGS in Pediatric BCP-ALL

#### 4.2.1. The Panel Choice

Before implementing NGS for clinical management of childhood leukemia, each center’s logistics, including available platforms, human, and economic resources, should be considered (Supplementary Figure S2). Additionally, comprehensive sequencing and integrative genome-wide analyses have profoundly refined the risk-stratification of pediatric BCP-ALL, resulting in the identification of new entities with prognostic and therapeutic significance [11]. Thus, the gene content may influence the panel choice and the clinical protocol should primarily guide this process.

Most commercially targeted NGS panels are designed as pan-cancer panels and contain a large number of genes. According to the literature, not all genes included in the panels have been shown to be clinically relevant, and, in the case that any clinically relevant gene was missed or not correctly covered, alternative methodologies should be performed to discard such abnormalities.

In this line, we have detected that any NGS panel is still facing at least two challenges in the BCP-ALL field: the detection of rearrangements, which is not fully covered in some currently available panels, and the detection of SNVs.

In this regard, the Human Comprehensive Cancer GeneRead v2^®^ covers a vast range of cancer-related genes, so it could be useful for those laboratories that study a wide span of cancers; however, in a clinical laboratory focused on a specific type of cancer, as hematological neoplasms, could not be the best option. On the other hand, the custom BCP-ALL, the OCCRA, and the Archer^®^ FusionPlex^®^ ALL panels were designed focusing on pediatric cancer and ALL. The core of genes shared by all panels for SNVs (*BRAF*, *CREBBP*, *CRLF2*, *EZH2*, *FLT3*, *IL7R*, *JAK1*, *JAK2*, *JAK3*, *KRAS*, *NF1*, *NRAS*, *PAX5,* and *PTPN11*) covered mainly those genes involved in new classification subtypes (such as *Ph-like* or *BCR-ABL1*-like, and *PAX5* alterations subtypes), which in turn represent new therapeutic targets involved in deregulated cell pathways in ALL [11,12,13,14]. Mutations in those genes related to BCP-ALL are pathogenically important and confer a better understanding of the disease. Thus, genetic testing might help clinicians tailoring personalized treatment to pediatric ALL patients. However, additional genes highly relevant to BCP-ALL were not included in the four panels design, such as *TP53*, *CSF1R,* or *NF1*. Therefore, when choosing an NGS panel for BCP-ALL, it might be important to prioritize the panel that includes the relevant genes with diagnostic, prognostic, and/or predictive value according to the current therapeutic protocols.

Interestingly, the main advantage of those panels that cover SNVs and fusion gene detection like the OCCRA panel (see Figure 3) is their ability to detect a wide percentage of abnormalities for accurate diagnostic classification, and the capacity to establish a precise patient risk stratification according to novel prognostic classification [13]. Of note, the Archer^®^ FusionPlex^®^ ALL open-ended PCR approach can detect previously unknown fusion genes and any partner of promiscuous genes; this is especially relevant in the BCP-ALL setting, as it allows to detect clinically relevant fusions involving *KMT2A* gene, as well as heterogeneous rearrangements involved in the Ph-like subtype.

Classical diagnostic techniques, such as karyotyping, PCR, and FISH, remain the gold standard in clinical routine in BCP-ALL diagnosis. Additionally, other conventional methods, such as MLPA and SNP-arrays, are often used to identify CNVs and larger chromosomal losses/gains. However, all the techniques mentioned above may miss identifying several alterations that have been recently reported by NGS approaches in the disease. Therefore, it is important to choose a panel covering a broad spectrum of BCP-ALL alterations, especially those new aberrations not detected by conventional methods.

#### 4.2.2. Technical Challenges of Each Analyzed Panel for BCP-ALL Diagnosis

Several methodological issues may be found in each step of targeted NGS. First is the sample and library preparation. In this regard, the BCP-ALL custom panel is laborious and time-consuming, in contrast with the Archer^®^ FusionPlex^®^ ALL, the easiest hands-on protocol of all the panels tested. The second is the sequencing and data analysis for variant detection. The user has to take into account if the chosen panel has technical support, like Archer^®^ FusionPlex^®^, or requires a specialist to develop a pipeline workflow. Another issue is the variant prioritization and reporting. Thus, we may find sequence errors due to artifacts that originate from library preparation, the sequencing process itself, or data analysis (e.g., read mapping, variant calling), resulting in misinterpretation of sequence variants [15]. Supplementary Table S9 summarizes the most significant challenges and possible approaches to tackle them.

Considering the hands-on in the optimization of our custom panel, we confirmed that the availability of commercial NGS panels allows an easier and faster application to the routine practice, as the panel performance has been previously tested and optimized by the company. However, even commercial panels need to be validated before their use in the clinical routine, as previously described.

Another issue to consider when choosing an NGS panel is the turnaround time expectation for the results, as it may play a crucial role in patient care. Considering the low incidence of pediatric BCP-ALL, the number of patients to analyze per week can be insufficient to perform a library each week. In this case, each laboratory should consider different options: (1) combining patients with different pathologies (BCP-ALL plus other types of leukemia, or solid tumor) using the same panel, as long as the scope of the panel allows it; (2) multiplexing different panels in the same run, or (3) using lower-capacity sequencing cartridges, studying only 1 or 2 patients per run. These approaches may allow shortening the turnaround time and providing the results in a clinically useful manner.

#### 4.2.3. Data Analysis for Variant Detection

Differences in data analysis with bioinformatics pipelines and variant prioritization are often ignored as a source of discrepancies. In this line, we observed some discrepancies due to different variant annotation and prioritization criteria used among centers. It is also important to emphasize that the harmonization of NGS panels must be accompanied by standardized bioinformatics algorithms to optimize inter-center reporting reproducibility. Some authors propose using bioinformatics pipelines independent of those offered by the sequencing platform manufacturers (often heterogeneous and poorly adapted to specific requirements) [16].

In this context, the genome sequence used as a reference for mapping and transcript for variant annotation is another crucial step [17]. Differences in the annotation for one variant may lead to discrepancies in the clinical results. An example is a variant found in *TP53* in sample 4 in our study. In this case, the use of different transcript sets resulted in the annotation for the same variant as intronic or exonic. This emphasizes the need for standardization of the annotation process to avoid the overstudy of potential disease-relevant variants and the omission of interesting variants incorrectly annotated. In the BCP-ALL setting, this issue is particularly relevant in some genes such as *TP53*, where mutations may occur throughout the gene [7].

#### 4.2.4. Variant Prioritization and Reporting

To harmonize the NGS workflow between different centers, the criteria for prioritizing the detected variants should also be considered. The filtering of germline variants, the use of different databases and cut-offs for total reads, and VAF reporting need to be agreed upon. Initially, we let each center use their analysis pipelines and variant prioritization criteria (setting only minimum common criteria) so that we could evaluate if these possible discrepancies truly exist in the results. With our study, we can assert the need for standardization in crucial steps and reach a consensus on the pipelines, reference genome, and variant prioritization criteria.

Finally, variant interpretation and reporting in the clinical context of the BCP-ALL remain a difficult task. Detected variants should be carefully reviewed by appropriately trained and certified molecular diagnostic professionals and integrated into the context of other clinical and laboratory findings (cytomorphology, immunophenotyping, cytogenetics, and molecular data). The molecular professional should report each variant using the current evidence derived from different American College of Medical Genetics and genomics/Associations of Molecular Pathology (ACMG/AMP) variant curation guidelines [18,19].

## 5. Conclusions: Lessons Learned and the Future Ahead

Previous publications have shown that NGS is a useful tool in the clinical context in the molecular characterization of leukemia [20,21,22]. In the era of personalized medicine, NGS may be useful, but not limited, to find targetable lesions in kinase-driven BCP-leukemias. It is crucial to assess the maximum number of potentially relevant molecular markers, as they could be used to refine the risk stratification and, if needed, to include patients in ongoing clinical trials with new drugs in different phases of the disease. Besides, the molecular MRD monitoring will probably expand in the future as more markers are arising in a clonal manner [23,24,25].

The application of NGS technology in clinical diagnostics should proceed with care. As there is no consensus yet on a universal methodology for NGS, a constant interaction between molecular experts, technicians, and clinicians is essential for selecting the right NGS panel at diagnosis, the sequencing methodology, and the correct interpretation of the NGS results. Moreover, the analytical validation of the chosen panel is a prerequisite for using NGS technology in clinical routine, both individually and among different centers in cooperative groups [26]. Our study highlights the importance of a comprehensive dissection of available NGS panels before implementing them into the clinics.

The interaction between clinical and molecular experts in our collaborative study amongst Spanish centers helped to define the minimum set of useful genes in pediatric BCP-ALL, fostered the collaborative research, and provided a step forward toward a consensus on NGS technologies, which will lead to their optimized use for the benefit of patients.

## Supplementary Materials

The following are available online at https://www.mdpi.com/2075-4426/10/4/244/s1, Figure S1: Overview of the study: workflow of samples, panels, and sequencing platforms, Figure S2: Application of different targeted-gene panels for BCP-ALL, Table S1: Main characteristics of the BCP-ALL patients included in the study, Table S2: List of the 69 genes included in the BCP-ALL custom panel, Table S3: Content of Archer^®^FusionPlex^®^ ALL Kit (ArcherDX, Inc., Boulder, CO, USA), Table S4: Content of OncomineTM Childhood Cancer Research Assay (ThermoFisher Scientific, Waltham, MA, USA), Table S5: List of genes of Human Comprehensive Cancer GeneRead Panel v2^®^ (Qiagen, Hilden, Germany), Table S6: Single-nucleotide variants reported by each center in the common samples after variant prioritization, using the BCP-ALL custom panel, Table S7: Single-nucleotide variants reported by each center in each of the common samples after variant prioritization, using the BCP-ALL custom panel and three commercial panels, Table S8: Fusion genes identified in the common samples analyzed with the commercial panels Archer^®^ FusionPlex^®^ ALL and OCCRA on different platforms, Table S9: Critical steps in NGS procedures: challenges and possible approaches to face them. Sample and library preparation.
