# Supplementary material for: Helpful Criteria When Implementing NGS Panels in Childhood Lymphoblastic Leukemia
Source: J Pers Med. 2020 Nov 26;10(4):244. doi: 10.3390/jpm10040244 (PMC7711852; doi:10.3390/jpm10040244)
Supplement: Supplementary file 1 [file jpm-10-00244-s001.pdf]

SUPPORTING INFORMATION

Collaborators

Supplementary Table 1. Main characteristics of the BCP-ALL patients included in the study.

Supplementary Table 2. List of the 69 genes included in the BCP-ALL custom panel.

Supplementary Table 3. Content of Archer®FusionPlex® ALL Kit (ArcherDX, Inc, Boulder, USA).

Supplementary Table 4. Content of Oncomine™ Childhood Cancer Research Assay (ThermoFisher Scientific, Waltham, Massachusetts, USA).

Supplementary Table 5. List of genes of Human Comprehensive Cancer GeneRead Panel v2® (Qiagen, Hilden, Germany).

Supplementary Table 6. Single-nucleotide variants reported by each center in the common samples after variant prioritization, using the BCP-ALL custom panel.

Supplementary Table 7. Single-nucleotide variants reported by each center in each of the common samples after variant prioritization, using the BCP-ALL custom panel and three commercial panels.

Supplementary Table 8. Fusion genes identified in the common samples analyzed with the commercial panels Archer® FusionPlex® ALL and OCCRA on different platforms.

Supplementary table 9. Critical steps in NGS procedures: challenges and possible approaches to face them. Sample and library preparation.

Supplementary Figure 1. Overview of the study: workflow of samples, panels and sequencing platforms.

Supplementary Figure 2. Application of different targeted-gene panels for BCP-ALL.

Supplementary Methods

Collaborators

The authors thank the contribution of other clinicians and researchers from different centers integrated in the Group of Leukemia of the Spanish Society of Pediatric Hematology and Oncology (SEHOP): Hospital Sant Joan de Déu (Albert Català, Roberta Malatesta, Camino Estella, Lídia Ruiz); Hospital Niño Jesús (Lorea Abad), Hospital Universitari i Politècnic La Fe (Inés Gomez-Seguí), Hospital Carlos Haya (Antonia Pascual); Hospital General de Alicante (Fabián Tarín, María Tasso); Hospital de Cruces (Marta Riñón, Rosa Adán); Hospital Virgen de la Arrixaca (Victoria Martinez-Sánchez, Mª Rosa Moya, José Luis Fuster); Hospital Vall d'Hebrón (Anna Varela, Cristina Díaz de Heredia); Hospital Virgen de las Nieves (Francisco Ruiz-Cabello, Pilar Jiménez, Mª José Ortega); Hospital Reina Sofía (Carmen Martínez); Hospital Virgen del Rocío (Clara García, Águeda Molinos, José Antonio Pérez-Simón); Hospital 12 de octubre (Estela Martín-Clavero, Pilar Guerra García); Hospital Gregorio Marañón (Carolina Martínez-Laperche, Marina García Morín); Hospital Taulí (Luz Muñoz, Sandra Pisa); Hospital Santa Creu i Sant Pau (Marta Pratcorona, Josep Nomdedéu, Isabel Badell); Hospital La Paz (Adela Escudero, Antonio Pérez-Martínez); Hospital Virgen de la Salud (Mª Rosario Velasco, Nerea Domínguez); Hospital Maternoinfantil de Badajoz (José Manuel Vagace); H.U Gran Canaria Doctor Negrín (Mª Teresa Gómez-Casares, Antonio Molinés); Hospital Marqués de Valdecillas (Mónica López-Duarte), Hospital de Donostia (Miriam García Abós); Complejo Hospitalario de Navarra (Reyes Robles, María Sagaseta de Ilurdoz); Hospital Virgen de la Macarena (David García, Helga Benítez, Ana Fernández-Teijeiro); Hospital Son Espases (Marta Bernués, Laia Ferrés, José Antonio Salinas).

Supplementary Table 1. Main characteristics of the BCP-ALL patients included in the study.

	Sample 1	Sample 2	Sample 3	Sample 4
**Age**	11.2	11.5	2.8	5.2
**Sex**	female	female	female	male
**Molecular subtype**	*BCR-ABL1*	Low hypodiploidy	B-other	*ETV6-RUNX1*
**Karyotype**	46,XX,?t(7;9),?t(9;12),t(9;22)(q34;q11)[22]/46,XX [3]	39,XX,-3,-7,-13,-15,-16,-17,-20[18]/46,XX[2]	46,XX,+9,add(9)(q?13),dic(9;20)(p11-13;q11)[22]/46,XX[2]	46,XY,del(6)(q?13q?23),add(9)(q34),del(12)(p13), del(13)(q?12q?14)(9)/47,idem,+?10(8)/46,XY(3)
**FISH**	• *BCR-ABL1* dual fusion*: BCR-ABL1* fusion found in 97% of nuclei	• No rearrangement found	• *PAX5* break apart: deletion in 33% of nuclei, no rearrangement	• *ETV6-RUNX1* dual fusion*: ETV6-RUNX1* fusion confirmed
**MLPA**	• SALSA P335: heterozygous deletion of *IKZF1 (exons 1-8), PAX5* and homozygous deletion of *CDKN2A/B*	• SALSA P335: heterozygous deletion of *IKZF1 (exons 1-8)* and *RB1*<br>• SALSA P181 & p182: loss of chromosomes 3, 7, 15,16,17 and 20	• SALSA P335: heterozygous deletion of *CDKN2A/B*	• SALSA P335: heterozygous deletion of *ETV6* and *EBF1*

Supplementary Table 2. List of the 69 genes included in the BCP-ALL custom panel.

**BCP-ALL custom panel**					
ADAMTS	*CDKN2B*	*EZH2*	*JAK1*	*MYC*	*RUNX1*
ADARB2	*CREBBP*	*FAT1*	*JAK2*	*NF1*	*SETD2*
APC	*CRLF2*	*FAT3*	*JAK3*	*NR3C1*	*SH2B3*
ASMTL	*CSF2RA*	*FLT3*	*KDM6A*	*NR3C2*	*TBL1XR1*
BLNK	*CTCF*	*HERC1*	*KMT2D*	*NRAS*	*TP53*
BRAF	*CTNNB1*	*HSPA13*	*KRAS*	*NT5C2*	*TSLP*
BTG1	*DYRK1A*	*IKZF1*	*LEF1*	*P2RY8*	*TYK2*
BTG3	*EBF1*	*IKZF2*	*MAPK1*	*PAX5*	*VPREB1*
BTLA	*EP300*	*IKZF3*	*MSH6*	*PEAK1*	*WHSC1*
CBL	*ERG*	*IL3RA*	*MTOR*	*PRPS1*	
CDKN1B	*ETS2*	*IL7R*	*MUC4*	*PTPN11*	
CDKN2A	*ETV6*	*ITGB2*	*MYB*	*RB1*	

Supplementary Table 3. Content of Archer®FusionPlex® ALL Kit (ArcherDX, Inc, Boulder, USA).

SNV/INDEL	EXPRESSION	FUSION	
ABL1	*ABL1*	*ABL1*	*PAG1*
BCL2	*AICDA*	*ABL2*	*PAX5*
BRAF	*BCL2*	*BCL11B*	*PBX1*
CREBBP	*BCL6*	*BCL2*	*PDCD1LG2*
CRLF2	*BLNK*	*BCL6*	*PDGFRA*
DNM2	*CD274*	*BCR*	*PDGFRB*
ETV6	*CRLF2*	*CHD1*	*PICALM*
EZH2	*CTLA4*	*CREBBP*	*PTK2B*
FBXW7	*DNTT*	*CRLF2*	*RUNX1*
FLT3	*FLT3*	*CSF1R*	*SEMA6A*
IDH1	*HOXA10*	*EBF1*	*SETD2*
IDH2	*HOXA9*	*EPOR*	*STIL*
IKZF3	*IRF4*	*ETV6*	*TAL1*
IL7R	*IRF8*	*FGFR1*	*TCF3*
JAK1	*LMO1*	*IKZF1*	*TYK2*
JAK2	*LYL1*	*IKZF2*	*ZCCHC7*
JAK3	*MYC*	*IKZF3*	
KDM6A	*PDCD1*	*JAK2*	
KRAS	*PDCD1LG2*	*KLF2*	
MPL	*PTPN1*	*KMT2A*	
NOTCH1	*RAG1*	*MLLT4*	
NRAS	*RAG2*	*MYC*	
NT5C2	*SOX11*	*NF1*	
PAX5	*TAL1*	*NOTCH*	
PDGFRA	*TLX1*	*NTRK3*	
PTPN11	*TLX3*	*NUP214*	
SH2B3	*WT1*	*NUP98*	
STAT3		*P2RY8*	
STAT5B			
TYK2			
WT1			

Supplementary Table 4. Content of Oncomine^{TM} Childhood Cancer Research Assay (ThermoFisher Scientific, Waltham, Massachusetts, USA).

COMPREHENSIVE MUTATION COVERAGE			FULL EXON COVERAGE		CNV	FUSION AND EXPRESSION			
ABL1	*FASLG*	*MYC*	*APC*	*NF1*	*ABL2*	*ABL1*	*JAK2*	*NTRK2*	*TFE3*
ABL2	*FBXW7*	*MYCN*	*ARID1A*	*NF2*	*ALK*	*ABL2*	*KAT6A*	*NTRK3*	*TP63*
ALK	*FGFR1*	*NCOR2*	*ARID1B*	*PHF6*	*BRAF*	*AFF3*	*KMT2A*	*NUP214*	*TSLP*
ACVR1	*FGFR2*	*NOTCH1*	*ATRX*	*PRPS1*	*CCND1*	*ALK*	*KMT2B*	*NUP98*	*TSPAN4*
AKT1	*FGFR3*	*NPM1*	*CDKN2A*	*PSMB5*	*CDK4*	*BCL11B*	*KMT2C*	*NUTM1*	*UBTF*
ASXL1	*FLT3*	*NRAS*	*CDKN2B*	*PTCH1*	*CDK6*	*BCOR*	*KMT2D*	*NUTM2B*	*USP6*
ASXL2	*GATA2*	*NT5C2*	*CEBPA*	*PTEN*	*EGFR*	*BCR*	*LMO2*	*PAX3*	*WHSC1*
BRAF	*GNA11*	*PAX5*	*CHD7*	*RB1*	*ERBB2*	*BRAF*	*MAML2*	*PAX5*	*YAP1*
CALR	*GNAQ*	*PDGFRA*	*CRLF1*	*RUNX1*	*ERBB3*	*CAMTA1*	*MAN2B1*	*PAX7*	*ZMYND11*
CBL	*H3F3A*	*PDGFRB*	*DDX3X*	*SMARCA4*	*FGFR1*	*CCND1*	*MECOM*	*PDGFB*	*ZNF384*
CCND1	*HDAC9*	*PIK3CA*	*DICER1*	*SMARCB1*	*FGFR2*	*CIC*	*MEF2D*	*PDGFRA*	
CCND3	*HIST1H3B*	*PIK3R1*	*EBF1*	*SOCS2*	*FGFR3*	*CREBBP*	*MET*	*PDGFRB*	
CCR5	*HRAS*	*PPM1D*	*EED*	*SUFU*	*FGFR4*	*CRLF2*	*MKL1*	*PLAG1*	
CDK4	*IDH1*	*PTPN11*	*FAS*	*SUZ12*	*GLI1*	*CSF1R*	*MLLT10*	*RAF1*	
CIC	*IDH2*	*RAF1*	*GATA1*	*TCF3*	*GLI2*	*DUSP22*	*MN1*	*RANBP17*	
CREBBP	*IL7R*	*RET*	*GATA3*	*TET2*	*IGF1R*	*EGFR*	*MYB*	*RARA*	
CRLF2	*JAK1*	*RHOA*	*GNA13*	*TP53*	*JAK1*	*ETV6*	*MYBL1*	*RECK*	**Gene Expression**
CSF1R	*JAK2*	*SETBP1*	*ID3*	*TSC1*	*JAK2*	*EWSR1*	*MYH11*	*RELA*	*BCL2*
CSF3R	*JAK3*	*SETD2*	*IKZF1*	*TSC2*	*JAK3*	*FGFR1*	*MYH9*	*RET*	*BCL6*
CTNNB1	*KDM4C*	*SH2B3*	*KDM6A*	*WHSC1*	*KIT*	*FGFR2*	*NCOA2*	*ROS1*	*FGFR1*
DAXX	*KDR*	*SH2D1A*	*KMT2D*	*WT1*	*KRAS*	*FGFR3*	*NCOR1*	*RUNX1*	*FGFR4*
DNMT3A	*KIT*	*SMO*	*MYOD1*	*XIAP*	*MDM2*	*FLT3*	*NOTCH1*	*SS18*	*IGF1R*
EGFR	*KRAS*	*STAT3*			*MDM4*	*FOSB*	*NOTCH2*	*SSBP2*	*MET*
EP300	*MAP2K1*	*STAT5B*			*MET*	*FUS*	*NOTCH4*	*STAG2*	*MYCN*
ERBB2	*MAP2K2*	*TERT*			*MYC*	*GLI1*	*NPM1*	*STAT6*	*MYC*
ERBB3	*MET*	*TPMT*			*MYCN*	*GLIS2*	*NR4A3*	*TAL1*	*TOP2A*
ERBB4	*MPL*	*USP7*			*PDGFRA*	*HMGA2*	*NTRK1*	*TCF3*	
ESR1	*MSH6*	*ZMYM3*			*PIK3CA*				
EZH2	*MTOR*								

Supplementary Table 5. List of genes of Human Comprehensive Cancer GeneRead Panel v2® (Qiagen, Hilden, Germany).

QIAGEN HUMAN COMPREHENSIVE CANCER GENEREAD PANEL V2®							
ABL1	*BUB1B*	*DDR2*	*FGFR2*	*IDH2*	*MEN1*	*PDGFRA*	*SMARCA4*
AKT1	*CARD11*	*DICER1*	*FGFR3*	*IKZF1*	*MET*	*PHF6*	*SMARCB1*
AKT2	*CBL*	*DNMT3A*	*FH*	*IL6ST*	*MLH1*	*PIK3CA*	*SMO*
ALK	*CBLB*	*ECT2L*	*FLCN*	*IL7R*	*MSH2*	*PIK3R1*	*SPOP*
AMER1	*CD79A*	*EGFR*	*FLT3*	*JAK1*	*MSH6*	*PMS2*	*SRC*
APC	*CD79B*	*EP300*	*FUBP1*	*JAK2*	*MTOR*	*PPP2R1A*	*STK11*
AR	*CDC73*	*EPCAM*	*GATA1*	*JAK3*	*MUTYH*	*PRDM1*	*SUFU*
ARID1A	*CDH1*	*ERBB2*	*GATA2*	*KDM6A*	*MYC*	*PRKAR1A*	*TERT*
ARID2	*CDK12*	*ERBB3*	*GATA3*	*KDR*	*MYD88*	*PTCH1*	*TNFAIP3*
ASXL1	*CDK4*	*ERBB4*	*GNA11*	*KIT*	*NF1*	*PTEN*	*TNFRSF14*
ATM	*CDKN2A*	*ERCC5*	*GNAQ*	*KLF6*	*NF2*	*PTPN11*	*TP53*
ATRX	*CHEK2*	*ESR1*	*GNAS*	*KMT2D*	*NFE2L2*	*RAC1*	*TSC1*
BAP1	*CIC*	*EZH2*	*GPC3*	*KRAS*	*NFKBIA*	*RB1*	*TSC2*
BCL6	*CREBBP*	*FAM46C*	*GRIN2A*	*MAP2K1*	*NOTCH1*	*RET*	*TSHR*
BCOR	*CRLF2*	*FANCA*	*H3F3A*	*MAP2K2*	*NOTCH2*	*ROS1*	*U2AF1*
BRAF	*CSF1R*	*FANCD2*	*HIST1H3B*	*MAP2K4*	*NPM1*	*SDHB*	*VHL*
BRCA1	*CTNNB1*	*FANCE*	*HNF1A*	*MAP3K1*	*NRAS*	*SETD2*	*WT1*
BRCA2	*CYLD*	*FAS*	*HRAS*	*MAP4K3*	*PALB2*	*SF3B1*	*XPC*
BRIP1	*DAXX*	*FBXO11*	*HSPH1*	*MDM2*	*PAX5*	*SLC7A8*	*ZNF2*
BTK	*DDB2*	*FBXW7*	*IDH1*	*MED12*	*PBRM1*	*SMAD4*	*ZRSR2*

Supplementary Table 6. Single-nucleotide variants reported by each center in the common samples after variant prioritization, using the BCP-ALL custom panel.

						Center 1		Center 2		Center 3	
Sample	SNV	Chromosome	cDNA	Protein	dbSNP ID	Total reads	VAF %	Total reads	VAF %	Total reads	VAF %
S1	*FAT1*	chr4:187584530	c.3503C>T	p.(Ser1168Leu)	rs200633985	714	45	30	36	333	49
	FAT3	chr11:92533057	c.6878A>G	p.(Asn2293Ser)	rs16918105	923	46	45	24	525	43
	FAT3	chr11:92535012	c.8833G>A	P.(Val2945Ile)	rs187454400	454	57	133	40	460	49
	ETS2	chr21:40177325	c.55G>A	p.(Ala19Thr)	rs73450548	1,009	48	248	40	-	-
S2	*MSH6*	chr2:48033484	c.3788G>A	p.(Arg1263His)	rs147852216 ;127593	282	53	239	47	260	54
	APC	chr5:112173839	c.2548A>G	p.(Arg850Gly)	-	267	49	-	-	103	52
	SH2B3	chr12:111856571	c.622G>C	p.(Glu208Gln)	rs202080221	108	48	103	51	126	51
	TP53†	chr17:7578211	c.638G>A	p.(Arg213Gln)	rs587778720	389	89	204	86	302	90
	NF1	chr17:29588751	c.4600C>T	P.(Arg1534*)	rs760703505	264	82	178	74	271	81
S3	*JAK2*	chr9:5126343	c.3188G>A	p.(Arg1063His)	rs41316003	173	94	162	91	432	93
	EP300	chr22:41521911	c.773C>T	p.(Thr258Ile)	rs763723986	418	61	264	45	417	46
	FLT3	chr13:28623587	c.970G>A	p.(Asp324Asn)	rs35602083	607	52	-	-	555	52
	FAT3	chr11:92526021	c.4700C>G	p.(Thr1567Ser)	rs192482905	-	-	219	38	-	-
	FAT3	chr11:92533735	c.7556G>A	p.(Arg2519Gln)	rs201507634	-	-	265	54	-	-
	FAT3	chr11:92564913	c.9607A<G	p.(Ile3203Val)	rs139337740	-	-	264	34	-	-
S4	*PTPN11*	chr12:112884118	c.53A>G	p.(Asn18Ser)	rs587778635	328	50	108	31	616	50
	ERG	chr21:39763629	c.844C>T	p.(Pro282Ser)	rs542821839	412	42	144	35	502	44
	TP53†	chr17:7576544	c.638C>T	p.(Ser213Leu)	rs758194998	-	-	81	37	-	-

SNV: single nucleotide variant. VAF: Variant Allelic Frequencies.
- : variant not detected
† The use of different transcript sets resulted in the annotation for the same variant as intronic or exonic.

Supplementary Table 7. Single-nucleotide variants reported by each center in each of the common samples analyzed with commercial panels.

	69-gene BCP-ALL custom panel	Oncomine™ Childhood Cancer Research Assay				Archer® FusionPlex® ALL (Illumina)				Human Comprehensive Cancer GeneRead Panel v2®			
	Variants found	Total reads	Reads R	Reads A	VAF %	Total reads	Reads R	Reads A	VAF %	Total reads	Reads R	Reads A	VAF %
Sample 1	*FAT1*	-	-	-	-	Regions not included				Regions not included			
	FAT3	-	-	-	-								
	FAT3	-	-	-	-								
	ETS2	-	-	-	-								
Sample 2	*MSH6*	-	-	-	-	Regions not included				223	114	108	48.4
	APC	1,999	1,029	970	48.52	-	-	-	-	1,334	714	614	46
	SH2B3	1,152	584	568	49.31	1,050	564	486	46.29	Regions not included			
	TP53	1,833	149	1,684	91.87	-	-	-	-	1,235	119	1,107	89.6
	NF1	1,943	337	1,566	80.6	-	-	-	-	Regions not included			
Sample 3	*JAK2*	1,998	129	1,869	93.64	-	-	-	-	977	65	910	93.1
	EP300	-	-	-	-	-	-	-	-	2,373	1,588	772	32.5
	FLT3	1,996	1,000	996	49.9	-	-	-	-	2,041	907	1,092	53.5
	FAT3	-	-	-	-	Regions not included				Regions not included			
	FAT3	-	-	-	-								
	FAT3	-	-	-	-								
Sample 4	*PTPN11*	-	-	-	-	34	17	17	50	2,558	1,156	1,391	54.4
	ERG	-	-	-	-	-	-	-	-	Regions not included			
	TP53	1,995	60	1,935	96.99	-	-	-	-	1512	832	671	44.4

Reads R: Reference reads; Reads A: Alternative reads; VAF: Variant Allelic Frequencies

- : variant not detected

***** Archer® FusionPlex® ALL (IonTorrent) not showed. IonReporter is not able to perform variant calling for this kit

Supplementary Table 8. Fusion genes identified in the common samples analyzed with the commercial panels Archer® FusionPlex® ALL and OCCRA on different platforms.

<table>
<tr><th rowspan="2"></th><th rowspan="2">Fusion genes</th><th rowspan="2">Genome position</th><th colspan="2">Archer® FusionPlex® ALL (Illumina)</th><th colspan="2">Archer® FusionPlex® ALL (IonTorrent)</th><th colspan="2">OCCRA</th></tr>
<tr><th>Rearrangement reads</th><th>Total reads*</th><th>Rearrangement reads</th><th>Total reads*</th><th>Rearrangement reads</th><th>Total reads*</th></tr>
<tr><td>Sample 1</td><td>BCR-ABL1</td><td>chr22: 23632600
chr9:133729451</td><td>8,313</td><td>1,265,524</td><td>1,821</td><td>1,012,327</td><td>245,820</td><td>1,153,129</td></tr>
<tr><td rowspan="3">Sample 2</td><td>LMO1-RIC3</td><td>chr11:8248522
chr11:8174970</td><td>44</td><td rowspan="3">1,453,990</td><td>19</td><td rowspan="3">863,879</td><td colspan="2" rowspan="3">Regions not included in OCCRA Panel</td></tr>
<tr><td>BCL11B-UNALIGNED-HOXA9</td><td>chr14;99737627
chr1:121
chr7:27204592</td><td>24</td><td>-</td></tr>
<tr><td>IKZF1</td><td>chr7:50367353
chr7:50467616Exon skipping</td><td>-</td><td>6</td></tr>
<tr><td>Sample 3</td><td colspan="8">No fusions detected</td></tr>
<tr><td rowspan="3">Sample 4</td><td>ETV6-RUNX1</td><td>chr12:12022903
chr21:36265260</td><td>3071</td><td rowspan="3">1,348,589</td><td>756</td><td rowspan="3">2,002,675</td><td>147,828</td><td rowspan="3">1,366,786</td></tr>
<tr><td>GIGYF2-PBX1</td><td>chr2:233568199
chr1:164661731</td><td>104</td><td>30</td><td>-</td></tr>
<tr><td>RUNX1-ETV6</td><td>chr21:36421139
chr12:12037379</td><td>-</td><td>837</td><td>-</td></tr>
</table>

OCCRA: Oncomine™ Childhood Cancer Research Assay; *Total reads per sample are listed

- : variant not detected

Supplementary table 9. Critical steps in NGS procedures: challenges and possible approaches to face them. Sample and library preparation.

	Challenge step	Background	Approach
**Sample preparation**	**Nucleic acid quality**	Bad quality DNA/RNA may result in poor quality libraries	It is better to work with freshly extracted DNA/RNA
	**Nucleic acid quantity**	Higher or Lower input of sample can affect the tagmentation process	Accurate quantification by Qubit
**Library preparation**	**Fragmentation**	Fragments longer or shorter than expected may lead to library loss	Highly recommended to perform a size control assay by 2100 Bioanalyzer
	**Pooling**	Different amounts of the different libraries will produce an unequal read depth of the libraries	Quantify the libraries by Qubit to adjust the volume used to pool libraries
**Sequencing and data analysis**	**Mapping genome reference**	The use of different reference genomes may lead to differences in the clinical impact of the variants	Choose carefully which transcript set and software will be used for annotation
	**Variant prioritization and annotation**	Not established criteria. Differences in bioinformatics pipelines and prioritization of the variants may result in discrepancies	It is important to standardize bioinformatics algorithms and the annotation
	**Discrimination of true genetic alterations**	Absence of validation of the variants may produce misleading results	Visualization of the aligned sequencing data and confirmation by Sanger sequencing when possible (VAF >15%)

Supplementary Figure 1. Overview of the study: workflow of samples, panels and sequencing platforms.

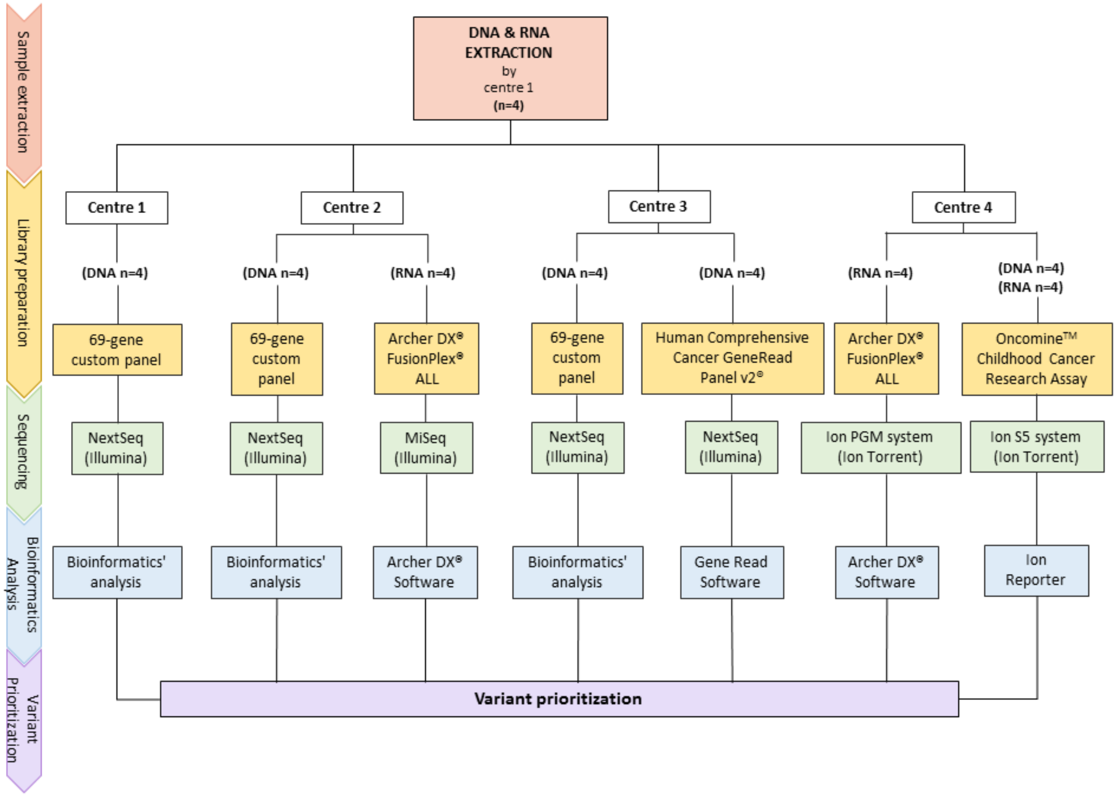

Supplementary Figure 2. Applicability of different targeted-gene panels for BCP-ALL.

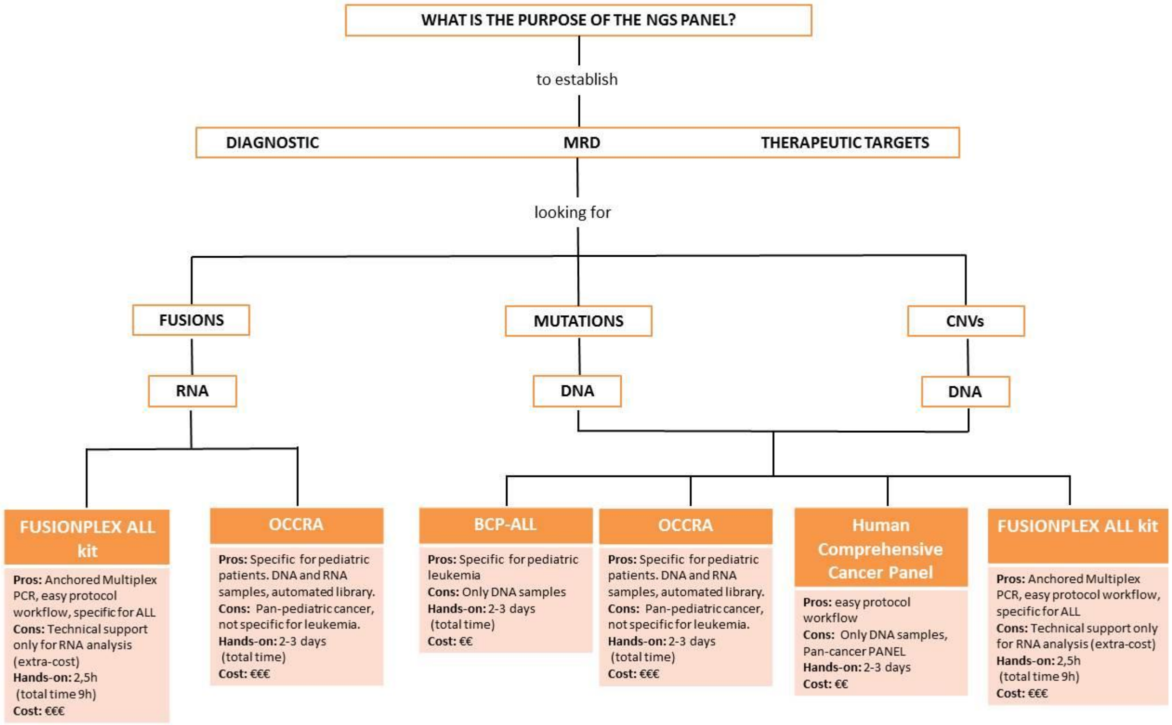

SUPPLEMENTARY METHODS

DNA and RNA extraction, quantification, and quality assessment

DNA and RNA were manually extracted in center 1 with the Gentra Puregene Kit (Qiagen, Hilden, Germany) and TriPure (Roche Diagnostics, USA), respectively. The DNA and RNA quality was assessed by agarose gel and by spectrophotometer (Quawell Q5000 UV-Vis Spectrophotometer, California, USA), having all the samples an OD260/280 ratio >1.8. The DNA and RNA were quantified using the Qubit dsDNA BR and HS Assay Kit and RNA BR and HS Assay kit, respectively, in a Qubit 4 Fluorometer (ThermoFisher Scientific).

BCP-ALL custom panel development

An exhaustive literature search was performed by clinical and molecular experts in ALL from the different centers, selecting those genes related to diagnostic, prognostic, or therapeutic interest recurrently (more than 1% of patients) identified in BCP-ALL.

The panel included 1,038 regions chosen in a restrictive approach covering only known hotspots. A total of 1,965 probes with an average size of 161 base pairs (bp) were designed. Exons were identified and flanked by up to 20 bases upstream and downstream the intronic sequence, to allow for the detection of variants occurring in conserved, proximal splicing elements. The cumulative target length was 197,077bp. Probe design and location were performed through Design Studio^{TM} (https://designstudio.illumina.com/), with 2% of probe overlap and the hg19/GRCh37 as the reference genome. The probes synthesis was performed by Illumina.

Library preparation and data analysis

Library preparation and subsequent sequencing runs were processed independently at each center, following the standardized workflows for each panel and instrument. Custom BCP-ALL libraries were generated and sequenced in 3 centers (centers 1, 2, and 3) on the Illumina

NextSeq500/550 Instrument, using a NextSeq500/550 High Output reagent kit v2 (300 cycles). The FusionPlex ALL® kit was sequenced on a MiSeq instrument (Illumina) (center 2) and on an Ion Torrent Personal Genome Machine (PGM) (center 4). The OCCRA was sequenced on an S5 sequencer at center 4. Finally, the Human Comprehensive Cancer GeneRead Panel v2® was sequenced on a NextSeq500/550 in center 3. Data analysis of the BCP-ALL custom panel was performed using in-house pipelines. For commercial panels, we used software provided by each company.

1) BCP-ALL custom panel

The BCP-ALL custom panel was assessed following the Nextera® Rapid Capture Enrichment protocol (Illumina). Based on amplicon concentration, equal amounts of amplicons were pooled to ensure that the optimal cluster density was achieved. After library pooling, two rounds of hybridization were performed, by binding targeted regions of the DNA with capture probes. Finally, size control was carried out with a 2100 Bioanalyzer (Agilent Technologies, California, USA) to ensure the library's good quality.

Bioinformatics pipeline for samples tested in Centre 1-HSJD

The variant detection protocol was developed at the Bioinformatics Unit from the Molecular Genetics Department at HSJD. The variant calling pipeline consisted of the following steps: first, read quality was assessed (FastQC v.0.11.5), low-quality adapters and reads were removed (Cutadapt v1.13) and remaining reads were aligned to the human reference genome (hg19/GRCh37) using Burrows-Wheeler Aligned through Bwa-mem v.0.7.15[1]. Reads with low mapping value and duplicates were removed (BEDtools v.2.26.0[2] and Picard v2.9.0). Variant calling was done with four different programs (SAMtools v.1.5[3], GATK v.3.7[4], FreeBayes v.1.1.0 and VarScan v.2.4.0[4]) and the annotation (SnpEff v.4.3[5]) included the gnomeAD, dbSNP, and Clinvar

databases. The pathogenicity values for each variant were determined by three predictors: SIFT[6], PolyPhen2[7] and MutationTaster[8].

Bioinformatic pipeline for samples tested in centers 2 & 3 (HUS and HNJ-ISCIII)

The first step was the *bcl* file generation, the base-calling which occurred within the sequencing instruments. After base-calling, the raw reads were generated in a FASTQ file format. Moreover, base-calling software supplied a quality metric for each base (Q) that indicates base-calling error probability. Usually, a quality threshold is set in a Q30, which means that the probability of the base being incorrectly called is 1 in 1000. The FASTQ files were analyzed by an in-house pipeline: 1) Trimmomatic v0.36, to remove bases with an insufficient quality and/or adapter; 2) FastQC y NGSQCToolkit v2.3.3, to evaluate sequencing quality and reads; 3) BWA v0.7.12 y GATK v3.5, to map the reads against the reference genome (GRCh37/hg19) and to mark PCR duplicates; 4) VarScan v2.3.9 and SAMTools v1.3.1, to detect variants and 5) ANNOVAR to annotate variants[9].

2) Archer® FusionPlex® ALL kit

For library generation, two different protocols were applied following the manufacturer´s specification depending on the sequencing platform (Illumina or Ion Torrent) used. In both cases, 250 ng of total input RNA was used. Libraries were quantified with the KAPA Universal Library Quantification Kit (Roche). Equimolecular libraries were pooled, amplified, and sequenced following standard procedures for Illumina MiSeq or Ion Torrent PGM sequencing. Results were analyzed on the Archer Analysis Software 6.0.

3) Oncomine™ Childhood Cancer Research Assay

Amplicon-based libraries for mutation and CNV detection were generated from 10ng of DNA following manufacturer specifications. For rearrangement and gene expression assessment, cDNA was obtained from 10ng of total RNA with the SuperScript™ VILO™ cDNA Synthesis Kit (ThermoFisher Scientific). Libraries were also generated according to the manufacturer's specifications. DNA and RNA libraries were quantified and diluted at 100pM, and further pooled at a 4:1 ratio (DNA:RNA). Emulsion PCR and chip load were carried out on an IonChef and sequenced on an S5 sequencer (ThermoFisher Scientific). Sequence alignment and variant calling were performed on the Torrent Suit 5.6 version. Variant annotation was carried out using the Ion Reporter software (5.6 version), applying the *Oncomine Childhood Cancer Research - w2.3 - DNA and Fusions - Single Sample* workflow. Aligned reads were also reviewed with Integrative Genomics Viewer 2.5 (Broad Institute and the Regents of the University of California)[10].

4) **Human Comprehensive Cancer GeneRead Panel v2® (Qiagen)**

Briefly, the amplicon libraries were prepared from 10ng of DNA according to the manufacturer's specifications and sequenced in a NextSeq500 sequencer (Illumina) in 2x150bp run format, to obtain an average depth of 1,000x reads. Raw data analysis for the identification of mutations was carried out with the GeneRead software, exon enrichment panel data analysis (Qiagen). Besides the automated analysis, the aligned files were visually reviewed using Integrative Genomics Viewer 2.5 (Broad Institute and the Regents of the University of California)[10].

Confirmation of variants identified by NGS

Sanger sequencing

Sanger sequencing was performed in patient-matched samples to confirm all the identified variants with ≥15-20% VAF. Different primer sets were designed using PRIMER3plus software

(https://primer3plus.com/cgi-bin/dev/primer3plus.cgi) and tested for all variants detected by the NGS panel. Sanger sequencing was performed using the BigDye Terminator v3.1 Cycle Sequencing Kit (ThermoFisher Scientific) and a 3130XL Genetic Analyzer (Applied Biosystems, TermoFisher Scientific).

Quantitative retro-transcriptase PCR (qRT-PCR)

Novel fusions detected by at least 2 independent RNA-based sequencing runs, and highly concordant alignments to the reference sequence were confirmed by q-RT-PCR. Specific primers were designed using the PRIMER3plus software (https://primer3plus.com/cgi-bin/dev/primer3plus.cgi). 500ng of total RNA was retrotranscribed with the High Capacity Retrotranscription kit (Applied Biosystems). Real-time PCR was performed as previously described[11] using GUS as a control gene. Primer sequences for *LMO-RIC1* fusion were LMO1-E2-F GCTCCACCCTCTACACCAAG and RIC1-E2-R CAGGAGTCTGGCCATCTGAG.

Confirmation of somatic nature of variants

To confirm the somatic nature of the variants identified by NGS, we used patient-matched bone marrow samples in complete morphological complete remission (CR) with negative or very low (<0.05%) measurable minimal residual disease (MRD), as assessed by 8-color flow cytometry or by quantitative PCR.

Confirmation of CNVs

CNVs were screened by MLPA using the SALSA MLPA P335 ALL-IKZF1, P181 and P182 kits (MRC Holland, Amsterdam, The Netherlands) according to the manufacturer's instructions.

References

1. Li H, Durbin R. Fast and accurate long-read alignment with Burrows-Wheeler transform. *Bioinformatics*. 2010;26(5):589-595. doi:10.1093/bioinformatics/btp698

2. Quinlan AR, Hall IM. BEDTools: A flexible suite of utilities for comparing genomic features. *Bioinformatics*. 2010;26(6):841-842. doi:10.1093/bioinformatics/btq033

3. Li H. A statistical framework for SNP calling, mutation discovery, association mapping and population genetical parameter estimation from sequencing data. *Bioinformatics*. 2011;27(21):2987-2993. doi:10.1093/bioinformatics/btr509

4. Wilson RK, Mardis ER, McLellan MD, et al. VarScan 2: Somatic mutation and copy number alteration discovery in cancer by exome sequencing. *Genome Res*. 2012;22:568-576. doi:10.1101/gr.129684.111

5. Cingolani P, Platts A, Wang LL, et al. A program for annotating and predicting the effects of single nucleotide polymorphisms, SnpEff. *Fly (Austin)*. 2012;6(2):80-92. doi:10.4161/fly.19695

6. Sim NL, Kumar P, Hu J, Henikoff S, Schneider G, Ng PC. SIFT web server: Predicting effects of amino acid substitutions on proteins. *Nucleic Acids Res*. 2012;40(W1):452-457. doi:10.1093/nar/gks539

7. Adzhubei IA, Schmidt S, Peshkin L, et al. A method and server for predicting damaging missense mutations. *Nat Methods*. 2010;7(4):248-249. doi:10.1038/nmeth0410-248

8. Schwarz JM, Cooper DN, Schuelke M, Seelow D. MutationTaster2: mutation prediction for the deep-sequencing age. *Nat Methods*. 2014;11(4):361-362. doi:10.1038/nmeth.2890

9. Bastida JM, Lozano ML, Benito R, et al. Introducing high-throughput sequencing into mainstream genetic diagnosis practice in inherited platelet disorders. *Haematologica*. 2018;103(1):148-162. doi:10.3324/haematol.2017.171132

10. Robinson JT, Thorvaldsdóttir H, Wenger AM, Zehir A, Mesirov JP. Variant Review with the Integrative Genomics Viewer. *Cancer Res*. 2017;77(21):e31-e34. doi:10.1158/0008-5472.CAN-17-0337

11. Dolz S, Barragán E, Fuster Ó, et al. Novel real-time polymerase chain reaction assay for simultaneous detection of recurrent fusion genes in acute myeloid leukemia. *J Mol Diagn*. 2013;15(5):678-686. doi:10.1016/j.jmoldx.2013.04.003
